# Vaccination with Recombinant Microneme Proteins Confers Protection against Experimental Toxoplasmosis in Mice

**DOI:** 10.1371/journal.pone.0143087

**Published:** 2015-11-17

**Authors:** Camila Figueiredo Pinzan, Aline Sardinha-Silva, Fausto Almeida, Livia Lai, Carla Duque Lopes, Elaine Vicente Lourenço, Ademilson Panunto-Castelo, Stephen Matthews, Maria Cristina Roque-Barreira

**Affiliations:** 1 Department of Cell and Molecular Biology, Ribeirão Preto Medical School, University of São Paulo, Ribeirão Preto, São Paulo, Brazil; 2 Division of Molecular Biosciences, Imperial College London, South Kensington Campus, London, SW7 2AZ, United Kingdom; 3 Department of Medicine, Division of Rheumatology, University of California Los Angeles, Los Angeles, California, 90095–1670, United States of America; 4 Department of Biology, School of Philosophy, Sciences and Literature of Ribeirão Preto, University of Sao Paulo, Ribeirão Preto, São Paulo, Brazil; Ehime University, JAPAN

## Abstract

Toxoplasmosis, a zoonotic disease caused by *Toxoplasma gondii*, is an important public health problem and veterinary concern. Although there is no vaccine for human toxoplasmosis, many attempts have been made to develop one. Promising vaccine candidates utilize proteins, or their genes, from microneme organelle of *T*. *gondii* that are involved in the initial stages of host cell invasion by the parasite. In the present study, we used different recombinant microneme proteins (TgMIC1, TgMIC4, or TgMIC6) or combinations of these proteins (TgMIC1-4 and TgMIC1-4-6) to evaluate the immune response and protection against experimental toxoplasmosis in C57BL/6 mice. Vaccination with recombinant TgMIC1, TgMIC4, or TgMIC6 alone conferred partial protection, as demonstrated by reduced brain cyst burden and mortality rates after challenge. Immunization with TgMIC1-4 or TgMIC1-4-6 vaccines provided the most effective protection, since 70% and 80% of mice, respectively, survived to the acute phase of infection. In addition, these vaccinated mice, in comparison to non-vaccinated ones, showed reduced parasite burden by 59% and 68%, respectively. The protective effect was related to the cellular and humoral immune responses induced by vaccination and included the release of Th1 cytokines IFN-γ and IL-12, antigen-stimulated spleen cell proliferation, and production of antigen-specific serum antibodies. Our results demonstrate that microneme proteins are potential vaccines against *T*. *gondii*, since their inoculation prevents or decreases the deleterious effects of the infection.

## Introduction

*T*. *gondii* is an obligate intracellular protozoan parasite that infects warm-blooded animals and causes toxoplasmosis. This wide host range makes *T*. *gondii* one of the most successful protozoan parasites. In pregnant women, the infection can lead to miscarriage, neonatal malformations, ocular complications, and severe cognitive impairment in the fetus [1, 2]. Furthermore, the infection can be fatal for immunocompromised patients such as those with AIDS [3, 4], organ transplant recipients [5, 6], or those with neoplastic disease [7]. In addition, toxoplasmosis can cause substantial economic losses to the farming industry [8, 9]. The most important interventions for toxoplasmosis rely on chemotherapeutic agents. However, the agents in use are inadequate, expensive, and often toxic [10, 11]. Until now, there is no commercial vaccine for use in humans, whereas a vaccine developed for veterinary use showed limited efficacy [12–14]. Therefore, the development of an effective vaccine or immunotherapy against human toxoplasmosis would be particularly valuable for preventing both primary fetal infection and reactivation in immunocompromised individuals. In addition, vaccination might reduce economic losses by preventing abortions in farm animals. Attenuated and inactivated parasites, genetically engineered antigens, and DNA vaccines are among potential vaccines for toxoplasmosis and have been tested for their immunological effects in animal models. Because of poor efficiency or biosafety concerns, only few vaccines have been licensed for use [15]. The characterization of molecules that play a role in the pathogenesis of *T*. *gondii* infection may constitute an important step in vaccine development.

Most of the studies performed on antigens involved in imparting protective immunity against *T*. *gondii* were focused on molecules that belong to 3 major protein families: surface antigens (SAGs), dense granule excreted-secreted antigens (GRAs), and rhoptry antigens (ROPs). However, the microneme proteins are particularly promising as vaccine antigens because they are responsible for host-cell recognition, binding, secretion of rhoptry organelles, and cell penetration of all apicomplexans [16–19]. Among the micronemes (MICs), *T*. *gondii* microneme protein 1 (*Tg*MIC1), *Tg*MIC4, and *Tg*MIC6 form a complex that exerts a very important role in host cell invasion [18]. We have previously reported that a lactose-affinity fraction (Lac+) purified from the soluble tachyzoite antigen of the *T*. *gondii* RH strain is constituted of TgMIC1 and TgMIC4, and that vaccination of C57BL/6 mice with Lac+ induces protective immunity against *T*. *gondii* [20, 21]. Such protection was demonstrated by increased survival rate and reduced tissue parasitism, as well as a Th1-specific immune response [20]. Yet the production of Lac+ from tachyzoites is an arduous task that involves several purification procedures and provides low protein yields, making unfeasible its use in vaccine development. Therefore, in the present work, we have generated recombinant TgMIC1, TgMIC4, and TgMIC6 proteins, which were evaluated individually or in several combinations for their ability to induce protective immunity in mice against infection by *T*. *gondii*.

## Materials and Methods

### 2.1. Animal ethics statement

All the experiments were developed in accordance to ethical principles in animal research adopted by Brazilian Society for Laboratory Animal Science and approved by the Ethics Committee on Animal Experiments, Ethical Committee of Ethics in Animal Research (CETEA) of the College of Medicine of Ribeirão Preto of the University of São Paulo (protocol number, 065/2012). All efforts were made to minimize animal suffering and the numbers of mice required for each experiment.

### 2.2. Mice and parasites

Female C57BL/6 (H-2^b^) mice aged 6–7 weeks were purchased from the animal house of the Campus of Ribeirão Preto, University of São Paulo. All mice were bred and maintained in small groups inside isolator cages with light/dark cycle of 12 hours, besides food and water ad libitum were provided, in the animal housing facility of School of Medicine of Ribeirão Preto, University of São Paulo. Cysts of the ME49 strain, which has low virulence for mice, were obtained from the brain of orally infected C57BL/6 mice, and maintained by monthly passage of 20 cysts per animal.

### 2.3 Preparation of soluble *T*. *gondii* antigens (STAg)

The tachyzoites of the RH strain obtained from the peritoneal exudate of infected mice were washed three times with phosphate-buffered saline (PBS, 10 mM sodium phosphate containing 0.15 M NaCl, pH 7.2) by centrifugation at 1,000 × *g* and suspended in PBS containing 0.8M phenyl methyl sulfonyl fluoride (Sigma Chemicals, St. Louis, USA). This parasite suspension was then sonicated (Vibra-cell; Sonics & Materials Inc., Danbury, USA) and centrifuged at 15,000 × *g* for 15 min, at 4°C. Supernatant was used as antigen source (STAg).

### 2.4. Isolation of lactose-binding proteins (Lac+)

Twenty milligrams of STAg was submitted to affinity chromatography on a 5-ml α-lactose-agarose column (Sigma Chemicals) previously equilibrated at 4°C with PBS containing 0.5 M NaCl. After washing the column with equilibrating buffer, the adsorbed material (Lac^+^) was eluted with 10 mL of 0.1M lactose in equilibrating buffer, concentrated, and dialyzed against water in an ultradiafiltration system using 10,000-Da cutoff membrane (YM10 -Amicon^®^ Division; W.R. Grace & Co., Beverly, USA).

### 2.5. Construction of the expression plasmid

A cDNA library from ME49-PDS *T*. *gondii* tachyzoites was kindly provided by Dr. Ian Manger, Department of Microbiology and Immunology, Stanford University School of Medicine (Stanford, CA, USA) and was used as the template for the initial PCR, to amplify the TgMIC1 and TgMIC4 genes, using the following specific primers: TgMIC1 5′(TCGCATTCTCATTCGCCGGCA)3′ and 3′(TCAAGCAGAGACGGCCGTAGGACT)5′ and for TgMIC4 5′(ATCACGCCTGCAGGTGATGAC)3′ and 3′(TCATTCTGTGTCTTTCGCTTCAAG)5′. These primers were designed on the basis of published sequences (GenBank accession numbers: TgMIC1—Z71786.1 and TgMIC4—AF143487.2). Two additional PCR steps have been performed for introducing the recombination sites attB1 and attB2 at both 5′—(5′-GGGGACAAGTTTGTACAAAAAAGCAGGC-3′) and 3′—end (5′-GGGGACC ACTTTGTACAAGAAAGCTGGG-3′) of the cDNA. The PCR products were cloned into a pDONR201 vector (Invitrogen, Carlsbad, CA) to obtain the pENTR-*Tg*MIC1 and pENTR-*Tg*MIC4 constructs used to transform DH5α *Escherichia coli* competent cells. The DNA from several Kan^r^ clones were sequenced. Inserts from pENTR-*Tg*MIC1 and pENTR-*Tg*MIC4 were transferred into pDEST17 vectors (Invitrogen) through a second recombination step and yielded pEXP17-*Tg*MIC1 and pEXP17-*Tg*MIC1. Plasmids were used to transform DH5α *E*. *coli* cells to select the Amp^r^ expression. Plasmids extracted from DH5α *E*. *coli* were transformed in *E*. *coli* BL21-DE3 competent cells to produce fusion proteins with N-terminal 6-histidine (6xHis) tag. The pET 21b plasmid containing full-length *Tg*MIC6 gene was kindly provided by Professor Stephen Matthews, Division of Molecular Biosciences, Imperial College London (UK).

### 2.6. Expression of *Tg*MIC1, *Tg*MIC4, and *Tg*MIC6 proteins

*E*. *coli* BL21 (DE3) (Novagen) cells transformed with pDEST17-MIC1, pDEST17-MIC4, or pET21b-MIC6 were grown on Luria-Bertani (LB) agar plates containing ampicillin (100 μg/mL) and chloramphenicol (34 μg/mL). Individual colonies were grown in 5 mL LB medium supplemented with ampicillin (100 μg/mL) at 37°C at 220 rpm. After 18 h, in 5 mL of LB medium supplemented with antibiotics. An aliquot of each culture was used to inoculate separately to 500 mL of fresh LB medium containing antibiotics. Cells were grown for 2–2.5 h until an optical density (OD_600_) of 0.4–0.6 was reached, when the expression was induced with the addition of 0.5 mM isopropyl-β-d-thiogalactopyranoside (IPTG). The culture was grown for additional 4 h and harvested by centrifugation at 4,500 × *g* for 20 min at 4°C.

### 2.7. Isolation of inclusion bodies using centrifugation

Cell pellets were suspended in disruption buffer consisted of 50 mM Tris-HCL pH 7.5 containing, NaCL 100mM, EDTA 5mM, PMSF 0,1 mM, 4 μl DNase (20,000 U) and lysozyme 1mg\mL. The cell pellets were stirred for 30 min at room temperature and disrupted by sonication (Sonics and Materials, Vibra cell) for 2 min × 10 (pulse on, 1 s; pulse off, 1 s; temperature of probe, 4°C; and amplitude, 80%). After ultra-sonication, the mixture was centrifuged at 15,200*g* for 15 min at 4°C. The pellet containing insoluble recombinant TgMICs proteins (inclusion bodies) were washed three times with 50 mM Tris–HCl, 100 mM NaCl, PMSF 0,1 mM and 0.5% Triton X-100 (pH 8.0), and finally with the same buffer without Triton X-100.

### 2.8. Inclusion body solubilization and refolding by gradient dialysis

The pellets were suspended in buffer containing 8 M urea, 50 mM glycine and 100 mM Tris–HCl, (pH 7.4) and solubilized overnight with stirring at room temperature, the solubilized inclusion body was centrifuged at 15,200*g* for 40 min at 4°C, and the supernatant was collected. Refolding was done with chaotropic agents’ concentration gradient dialysis using a previously described method, with modifications [22]. The solutions of denatured proteins were dialyzed against 2 L of freshly made 4, 2, 1, 0.5, and 0 M urea, respectively, with 100 mM Tris-HCl (pH 7.4). With each concentration, the protein was dialyzed 24 h at 4°C. After the urea removal the samples were dialyzed against 5 mM Tris-HCl (pH 7.4) an ultradiafiltration system using 10,000-Da cutoff membrane (YM10 -Amicon^®^ Division; W.R. Grace & Co., Beverly, USA).

To remove endotoxin impurities, endotoxin-free columns (Bio_Rad, 0,5x10 cm) were packed with 1ml polymyxine B suspended in pyrogen-free water. The microneme proteins fractions were pumped over the columns with a flow rate of 1 ml/min and collected in sterile pyrogen-free tubes. The endotoxin level was measured with a Chromogenic End-point Endotoxin Assay Kit (Chinese Horseshoe Crab Reagent Manufactory, Xiamen, China). Less than 0.1 EU/ml of endotoxin was detected in the final protein preparations.

### 2.9. Immunization

C57BL/6 mice were subcutaneously (s.c.) injected with *Tg*MIC1 (10 μg), TgMIC4 (10μg), TgMIC6 (10μg), TgMIC1-4 (5 μg of each protein), TgMIC1-4-6 (3.3μg of each protein), or Lac+ (10 μg) emulsified in Freund’s complete adjuvant (Sigma Chemicals). Animals were boosted at the same dose and regimen on day 15 and 30 after first injection, now emulsified in Freund’s incomplete adjuvant (Sigma Chemicals). A control group was injected at the same regimen with PBS emulsified in Freund’s adjuvant (vehicle). Fifteen days after the last injection, blood and spleen samples were collected to assess serum IgG, *in vitro* T cell proliferation, and cytokine concentrations.

### 2.10. Determination of *T*. *gondii-*specific IgG and IgG subclass titers

Specific antibody responses were analyzed by enzyme-linked immunosorbent assay (ELISA). For total IgG detection, the collected serum samples were diluted 1:25, 1:50, 1:100, 1:200, and 1:400. For isotype detection, the serum samples were diluted 1:100. The assays were performed in microtiter plates (Nunc, Naperville, USA), which were coated with STAg (50 μL per well) at a concentration of 10 μg/mL in 50 mM sodium carbonate buffer, pH 9.6, overnight, at 4°C. The plates were then washed three times in PBS containing 0.05% Tween 20 (PBS-T) (pH 7.4). Non-specific binding sites were blocked with PBS-T containing 1% bovine serum albumin (BSA; blocking buffer), for 1 h, at 37°C. Serum samples (50 μl) were added to duplicate wells in blocking buffer. Plates were then incubated at 37°C for 1 h, washed four times, and incubated with horseradish-peroxidase-conjugated goat anti-mouse IgG, IgG1, or IgG2b (Santa Cruz Biotechnology), at 1:5000 dilution in blocking buffer, for 1 h, at 37°C. After washing with PBS-T, reactions were developed with the TMB (3,30,5,50-tetramethylbenzidine) Substrate Kit, according to the manufacturer’s instructions (Pierce Chemical Co., Rockford, USA). The reaction was stopped 15 min later by addition of 25μL of 1M sulfuric acid to each well. The absorbance was read at 450 nm by using a Microplate Scanning Spectrophotometer (PowerWavex; Bio-Tek Instruments, Inc., Winooski, USA).

### 2.11. Cytokine level quantification

The spleens aseptically removed from mice, fifteen days after the last immunization procedure, were gentle disrupted and passed through nylon mesh in RPMI medium. Single-cell suspensions were depleted of erythrocytes by hypotonic shock, and suspended in RPMI medium (Sigma Chemicals) supplemented with 10% fetal calf serum, 10 mM HEPES, 2 mM l-glutamine, 1 mM sodium pyruvate, and 50μM β-mercaptoethanol. The cells were then seeded in flat-bottom 24-well microtiter plates (Corning, USA) at density of 1 × 10^6^ cells per well, in 1 mL of the culture medium alone or medium with STAg (10 μg/mL) in duplicate. Cells were stimulated with concanavalin A (2 μg/mL) as positive control. The plates were incubated in atmosphere containing 5% CO_2_ at 37°C. The optimal STAg concentration was previously determined by a dose response assay. Cell-free supernatants were harvested and assayed for IL-12p40 and IL-4 concentrations following a 24-h culture, IL-10 concentration, 72-h culture, and IFN-γ concentration, 96-h culture. Cytokine production in supernatants was quantified using an ELISA kit, according to the manufacturer’s instructions (OptEIA set; Pharmingen, San Diego, CA).

### 2.12. Lymphoproliferation assay

Spleen cells, prepared as described previously, were suspended in RPMI medium (Sigma Chemicals) supplemented with 10% fetal calf serum, 10 mM HEPES, 2 mM l-glutamine, 1 mM sodium pyruvate, and 50μM β-mercaptoethanol. They were distributed in flat-bottom 96-well microtiter plates (Corning) at a density of 5 × 10^5^ cells per well, in 200 μL of culture medium alone or medium with STAg (10 μg/mL) in triplicate. Cells were stimulated with concanavalin A (2 μg/ml) as positive control. The plates were incubated for 3 days in atmosphere containing 5% CO_2_ at 37°C and pulsed with 1 μCi [^3^H]thymidine per well for an additional 18-h period. The cells were next harvested onto glass fiber filters and incorporated radioactivity (counts per minute [cpm]) was measured in a liquid scintillator. The results are expressed as mean counts per minute (count) for three replicates.

### 2.13. Challenge and infection

One month after the last immunization procedure, the mice were orally infected with 80 cysts of the ME49 strain and were monitored and recorded for 30 days to compute the survival rates. During this period, the infected mice were closely monitored two times a day (at 8 am and 6 pm) for their clinical appearance. The mice were weighed daily and monitored for clinical signs of infection–weight loss, ruffled fur, hunched posture, decrease in appetite, weakness/inability to obtain feed or water, lethargy and morbidity. If obvious sufferings were observed, the mice were immediately euthanized using CO_2_ in a custom flow metered chamber. At the end of the experimental window, all the remaining mice were euthanized with CO_2_.

To evaluate the effect of immunization on tissue cyst burden, the brain of the mice infected with 40 cysts was removed 1 month after the challenge and homogenized in 1 mL of PBS, by passing it through a 25-×-8-gauge needle (Becton Dickinson, Curitiba, Brazil). Cysts were separated from the brain tissue by using a previously described method, with modifications [23]. Homogenates were centrifuged in 16% dextran gradient (industrial grade, average molecular weight: 170,000; Sigma Chemicals) in PBS, at 3,000 × *g* for 15 min at 4°C. The mean number of cysts per brain was determined by phase-contrast microscopy at 100× magnification and counting five samples (8 mL each) of each pellet. The results are expressed as means ± SD for each group.

Additionally, blood samples from groups of 4 infected mice were collected 30 days after *T*. *gondii* challenge and the serum cytokine levels were analyzed by ELISA as described above. Blood samples from these mice were collected after the injection with anesthetics Ketamine (Syntec Brasil Ltda, SP, Brazil)/Xylazine (Schering-Plough Coopers, SP, Brazil), (10/0.5 mg per 100 g body weight) by i.p. route and then the mice were euthanized with CO_2_.

### 2.14 Statistical analysis

Statistical determinations of the difference between means of experimental groups were performed using one or two-way analysis of variance (ANOVA) followed by Bonferroni’s post-test using GraphPad Prism software (GraphPad Software, San Diego, CA, USA). Values were considered significant when p <0.05. All experiments were performed at least three times.

## Results

### 3.1. Expression and purification of TgMIC1, TgMIC4, and TgMIC6 recombinant proteins

**Fig 1:**
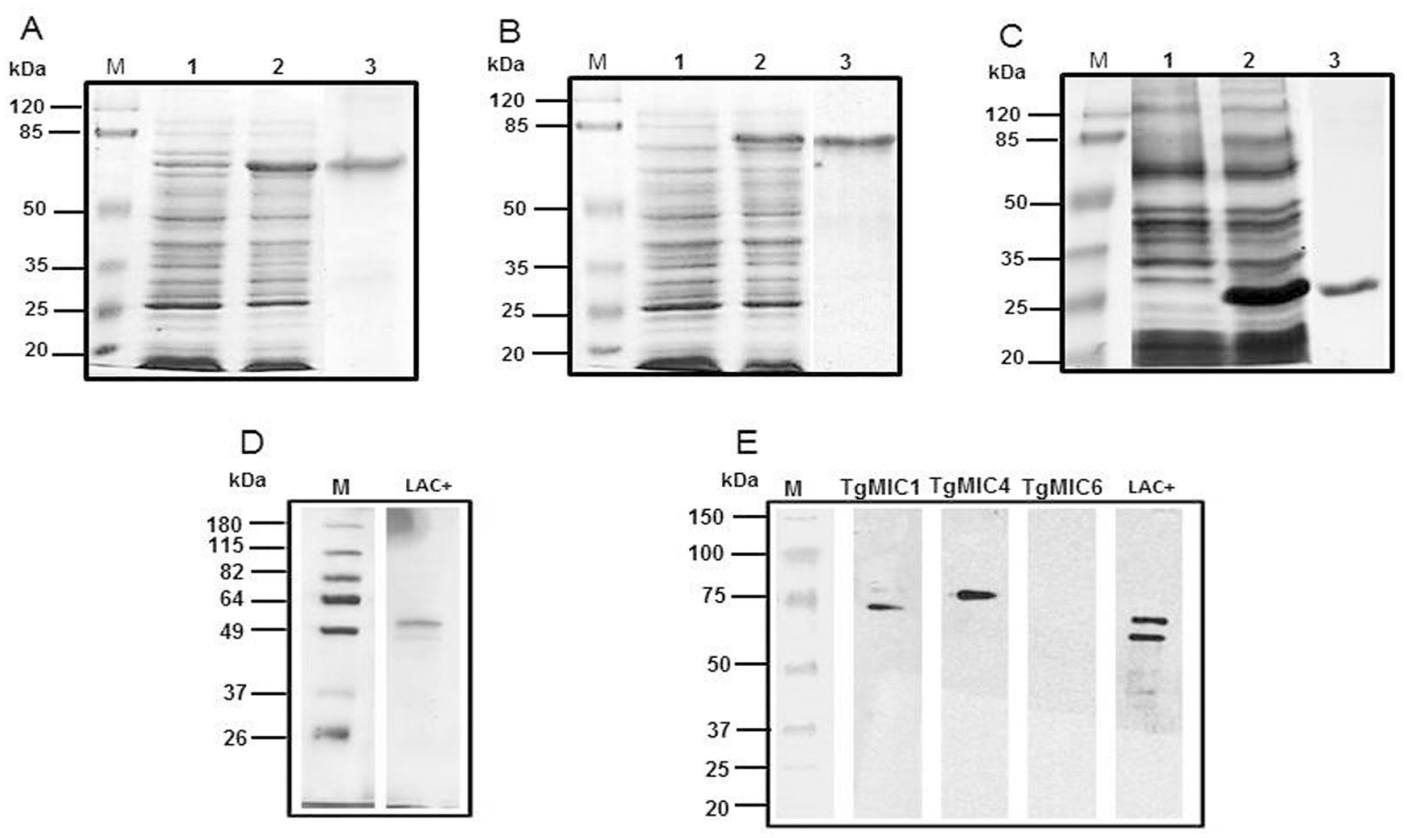
SDS-PAGE and western blot analysis of native and recombinant microneme proteins. SDS-PAGE of recombinant proteins (panels A, B, and C, Coomassie Blue stained) or native (panel D, silver-stained) proteins. Heterologous expression was noted in *E*. *coli* (DE3) and recombinant proteins were detected in inclusion bodies. Lane 1: protein expression before induction. Lane 2: protein expression after induction. Lane 3: purified and refolded histidine-tagged recombinant proteins, displayed apparent molecular masses of 70-kDa (TgMIC1, panel A), 80-kDa (TgMIC4, panel B), and 30-kDa (TgMIC6, panel C). Panel E shows the electrophoretical profile of the Lac+ fraction, composed of the native proteins TgMIC1 (53-kDa) and TgMIC4 (68-kDa). Lane M: Molecular mass markers. Reactivity of recombinant and native microneme proteins with anti-Lac+ mouse serum was examined by western blot (Panel E), developed with peroxidase-conjugated goat anti-mouse IgG.

Fig 1 shows the electrophoretic profile of samples harvested from recombinant clone cultures before (lane 1) and after induction (lane 2) for the expression of TgMIC1 (panel A), TgMIC4 (panel B), and TgMIC6 (panel C). The *T*. *gondii* microneme proteins expressed in *E*. *coli* were insoluble and contained inclusion bodies. They were diluted in refolding buffer containing 8M urea, until concentrations as low as 100μg/ml were reached. Sequential dialysis removed the denaturing agent and provided high yields of soluble proteins. SDS-PAGE of the purified recombinant proteins showed a single band for each preparation, with molecular masses of 70-kDa for TgMIC1, 80-kDa for TgMIC4, and 30-kDa for TgMIC6 (Fig 1, lane 3 of Panels A, B, and C). These masses are compatible with the predicted molecular mass for each protein together with a polyhistidine-tag. The immunoreactivity of TgMIC1 and TgMIC4 preparations with anti-Lac+ mouse serum was confirmed by western blot analysis; however, no reactivity was observed with TgMIC6 preparation (Fig 1, panel E). The Lac+ fraction provided two major protein bands with molecular masses of 53 and 68-kDa (Fig 1, panel D), both of which were identified in the serum sample from anti-Lac+ mice (Fig 1, panel E). This recognition indicates that the recombinant TgMIC1 (rTgMIC1) and TgMIC4 (rTgMIC4) had antigenic similarities with the corresponding native proteins. The lack of rTgMIC6 recognition by anti-Lac+ serum was predictable, since this microneme protein is not a constituent of the Lac+ fraction purified from the parasite, as already demonstrated and now reinforced by the electrophoretic profile of Lac+ shown in Fig 1, panel D.

### 3.2. Humoral immunity elicited by vaccination with microneme proteins

**Fig 2:**
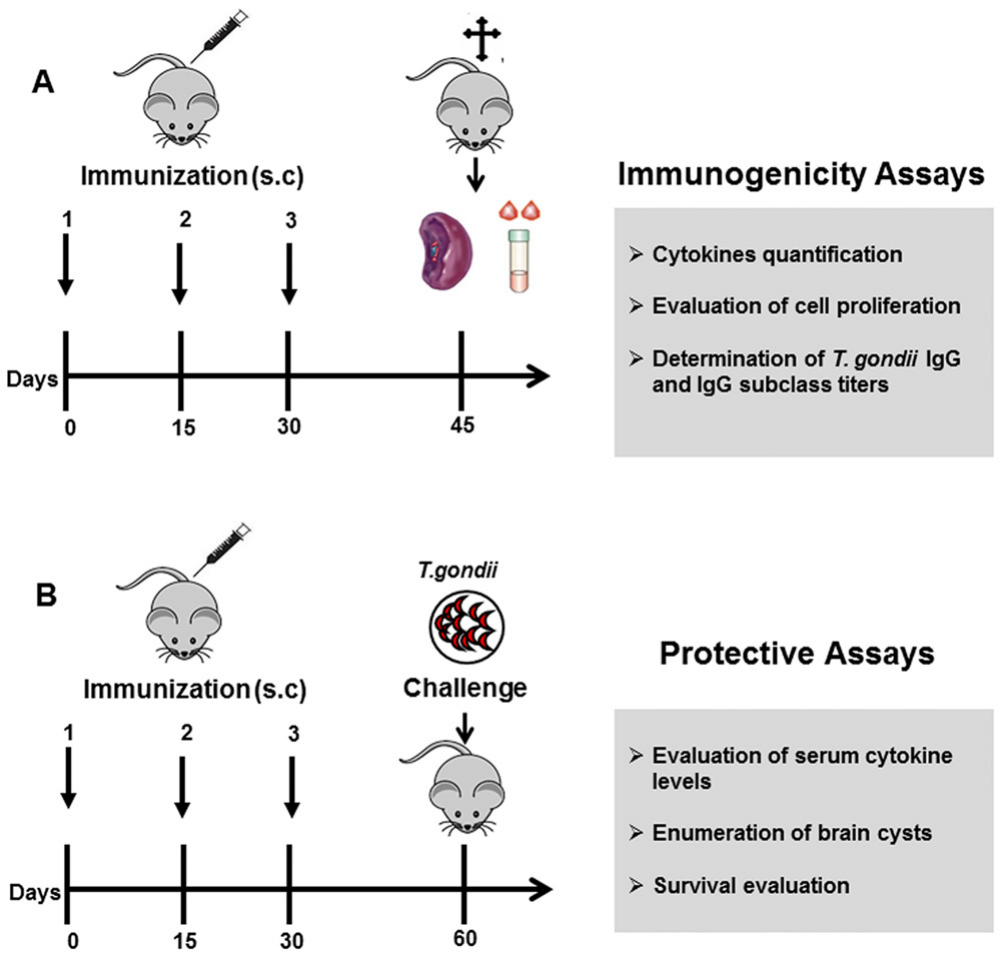
Experimental Protocol. **(A)** In the first experimental procedure mice were subcutaneously (s.c.) vaccinated with microneme proteins emulsified in Freund’s complete adjuvant. Mice were boosted at the same dose and regimen on day 15 and 30 after first injection, now emulsified in Freund’s incomplete adjuvant. Fifteen days after the last injection, blood and spleen samples were collected to assess serum IgG, *in vitro* T cell proliferation, and cytokine concentrations. **(B)** One month after the last immunization procedure, the mice were orally infected with 80 cysts of the ME49 strain and the mortality was monitored daily for 1 month. To evaluate the tissue cyst burden, the brain of the mice infected with 40 cysts was removed 1 month after the challenge and the mean number of cysts per brain was determined. Additionally, blood samples from mice challenged with 40 cysts were collected 30 days after *T*. *gondii* challenge and the serum cytokine levels were analyzed.

**Fig 3:**
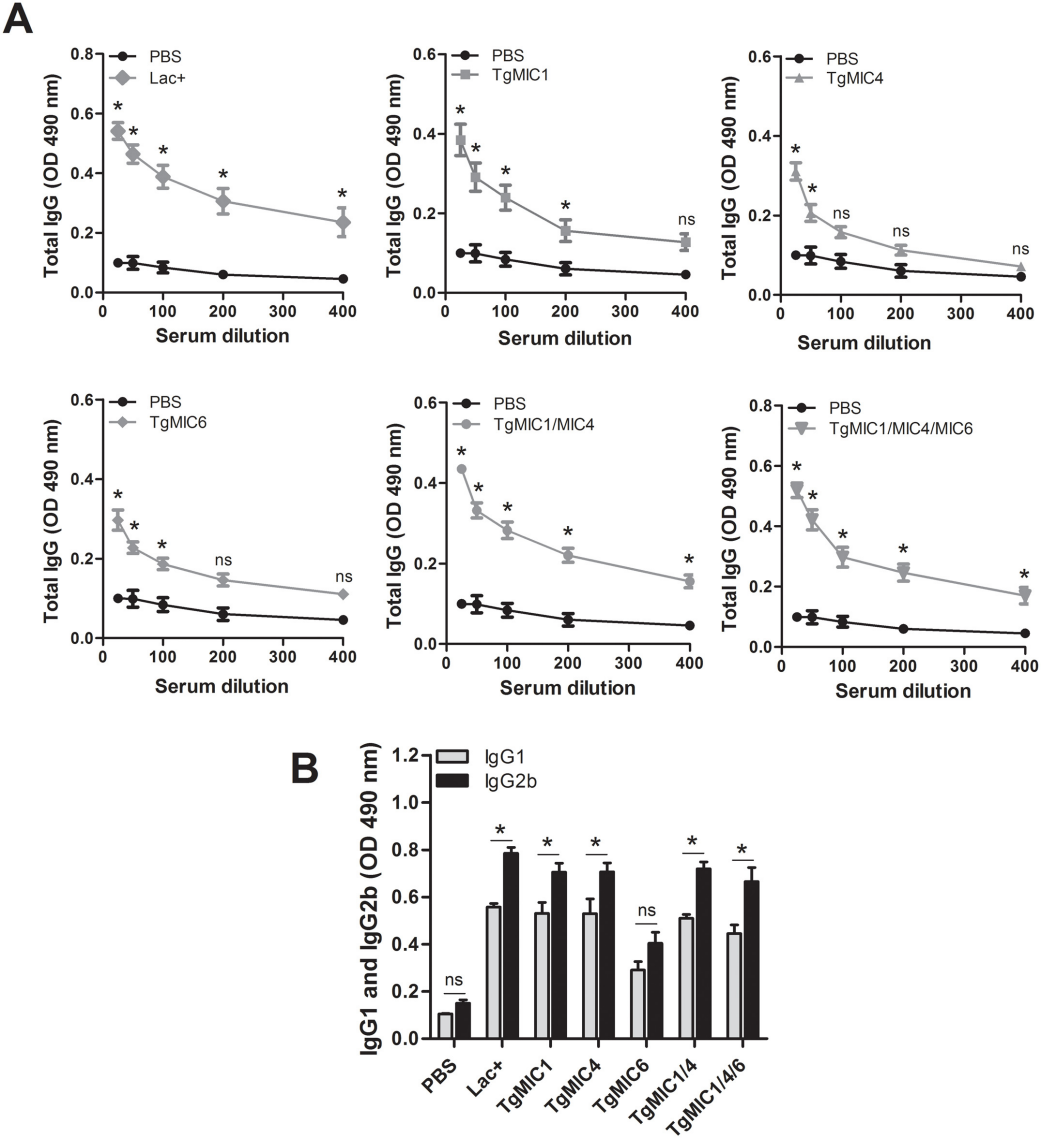
Specific humoral responses elicited by immunization of mice with microneme proteins. The reactivity of immunoglobulins anti-STAg was determined by ELISA in serum samples collected from both immunized (TgMICs) and control (PBS) mice 15 days after the last antigen injection. Each point/bar represents the average absorbance ± SD of the serum samples from 4 animals. (A) Absorbance provided by the reaction of serum IgG with STAg. (B) Absorbance provided by the reaction of serum IgG1 and IgG2a (diluted 1:25) with STAg. The average absorbance ± SD generated by the reaction of serum IgG1 or IgG2a from each group of immunized mice was significantly higher than the corresponding values provided by control mice, with the exception of the TgMIC6-immunized group, whose results were not significantly different of those of the control group. Three independent experiments were performed, and data from one representative experiment is shown. Asterisks represent statistical significant differences (*p < 0.05) between IgG2b and IgG1 for each group of mice (Bonferroni’s t test).

To evaluate whether immunization protocol (Fig 2) with microneme proteins could elicit specific humoral responses, serum samples from immunized and control mice were collected fifteen days after the last booster, and specific IgG levels were analyzed by ELISA. High IgG titers were detected in the sera of all immunized mice. Notably, the titers were higher in the sera from the mice immunized with combinations of microneme proteins, i.e., TgMIC1-4, TgMIC1-4-6, and Lac+ than in the sera from the mice immunized with individual proteins, i.e., TgMIC1, TgMIC4, or TgMIC6 (Fig 3A). We also examined whether a skew toward Th1 or Th2 immunity could be inferred from the levels of specific IgG1 and IgG2b subclasses detected in the serum samples. Immunized mice in all groups presented higher serum levels of specific IgG1 and IgG2b, in comparison to the sera of the non-immunized mice (Fig 3B). A significantly higher IgG2b value than IgG1 was observed in mice immunized with combinations of recombinant microneme proteins (TgMIC1-4, TgMIC1-4-6) or with the native complex Lac+ (obtained from STAg and comprising TgMIC1-4), while no significant differences were observed in the control group (PBS), suggesting that these microneme proteins elicited prominent Th1 immunity.

### 3.3 Cellular immunity elicited by vaccination with microneme proteins

**Fig 4:**
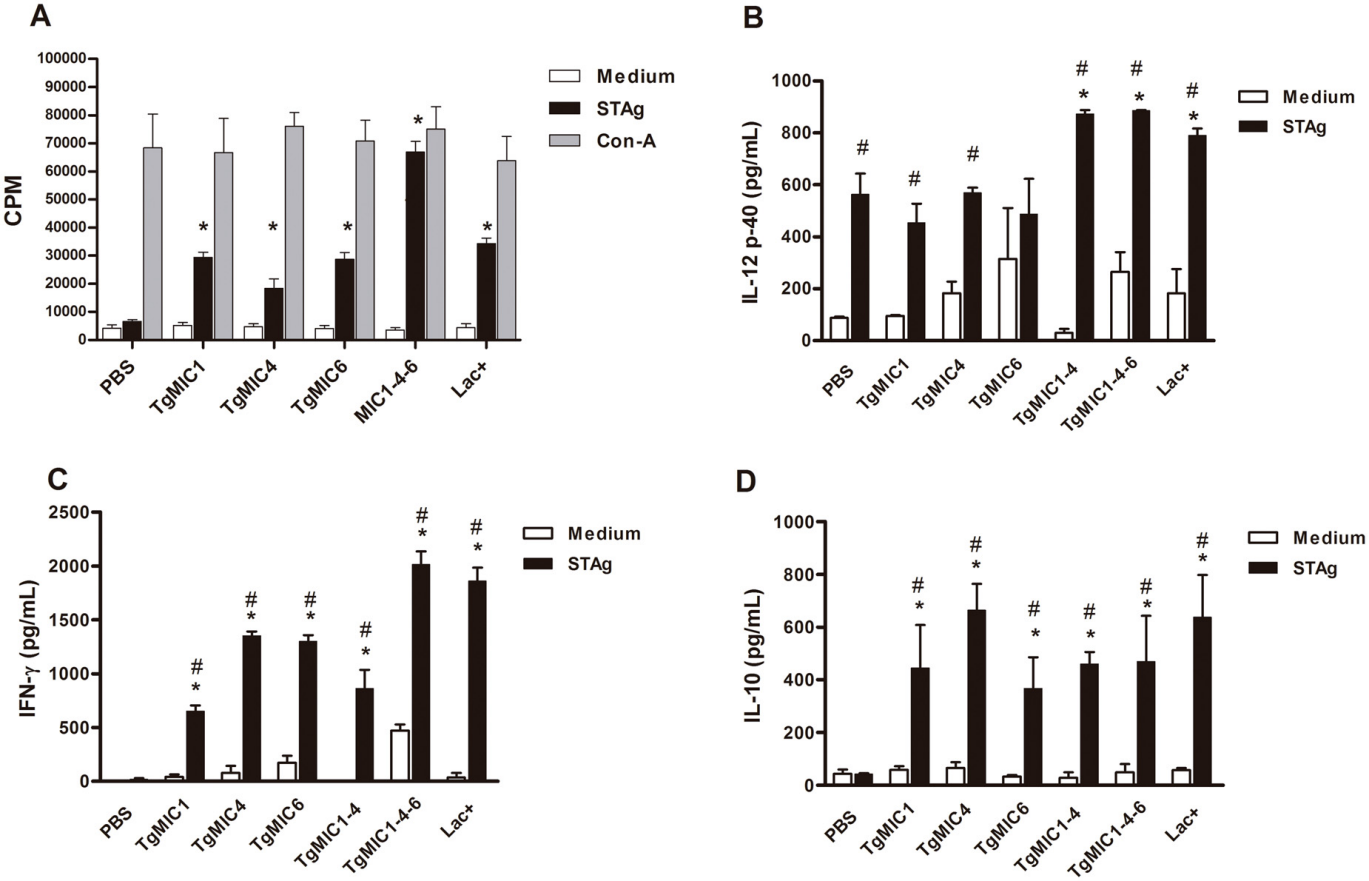
Specific cell-mediated immune responses elicited by immunization with microneme proteins. Spleen cells were harvested from immunized (indicated as TgMICs) and control mice (PBS) on day 15 post the last antigen injection and cultured in the presence of medium only, STAg (10 μg/ml), or Concanavalin A (2 μg/ml) for 72 h. (A) Proliferation of spleen cells was measured by [3H]-thymidine incorporation assay. Each bar represents the average of four mice per group and is representative of three independent experiments. Statistical significance is denoted as *p < 0.05 compared to the control group. (B–D) Cytokine concentration was measured by ELISA in the supernatant of spleen cell cultures. Panels show the IL-12 (B), IFNγ(C), and IL-10 (D) concentrations. Each bar represents the mean ± SD of triplicate samples and the results are representative of three independent experiments. Statistical significance is denoted as *p < 0.05 compared to PBS-inoculated mice; # p < 0.05 compared to non-stimulated cells of the same group.

We compared the proliferative response of the spleen cells from vaccinated and non-vaccinated animals stimulated *in vitro* with STAg. Proliferation of the cells from immunized mice was significantly higher than that of the cells from non-immunized mice. Cell proliferation was at least 2 times higher in the spleen cells from the mice immunized with TgMIC1-4-6 compared to that observed in cells from other groups of immunized mice, which was comparable to cell proliferation from any group of mice polyclonally stimulated with Con A (Fig 4A).

The cell-mediated immunity generated by the immunized mice was also evaluated by measuring the levels of cytokines (IL-12p40, IFN-γ, IL-4, and IL-10) released in the supernatant of spleen cells cultured under STAg stimulation (Fig 4B, 4C and 4D). Spleen cells from all groups of immunized mice displayed high IL-12 production, primarily those from mice immunized with multicomponent preparations, because they produced twice as much IL-12 than the cells from mice immunized with single-component microneme protein. Notably, STAg stimulation generated IL-12 production by the spleen cells from non-immunized mice (PBS), in amounts that were close to those released by cells from mice immunized with single-component preparations such as TgMIC1, TgMIC4, or TgMIC6. High concentrations of IFN-γwere predominantly produced by cells of all groups of immunized mice, especially by those immunized with multicomponent preparations, such as TgMIC1-4, TgMIC1-4-6 and Lac+. High levels of IL-10 were also produced by STAg-stimulated spleen cells from all groups of immunized mice. There was no difference in the IL-10 production pattern displayed by cells from mice that were immunized with single- or multi-component preparations. IL-4 was not detected in any culture supernatant (data not shown). It is noteworthy that in cells from non-immunized mice, STAg stimulated only IL-12 production.

### 3.4 Protection conferred by immunization with microneme proteins

Since our results indicate that immunization with microneme proteins elicits Th1 response, which is the type required for the development of resistance to toxoplasmosis (reviewed in ref. [24]), we investigated if the vaccination procedure could confer protection to *T*. *gondii* infection. The serum cytokine levels on day 30 post the challenge with *T*. *gondii* cysts were determined (Table 1).

**Table 1:** Serum cytokine levels of mice immunized with microneme proteins and infected with *T*. *gondii*. Cytokine levels in the serum of mice immunized on days 0, 15, and 30 with the indicated preparations of TgMICs or with vehicle (PBS), and after one month (day 60), challenged with *T*. *gondii* infection, provoked by gavage with 40 cysts of the ME49 strain. Cytokine levels were determined by ELISA in samples collected one month after challenge (on day 90).

Groups	Cytokine (ng/ml)			
	IL-12	IFN-γ	IL-4	IL-10
PBS	1.8±0.41	0.53±0.17	2.0±0.1	0.7±0.15
TgMIC1	5.4±1.1	1.6±0.57	1.2±0.14 *	1.0±0.54
TgMIC4	4.9±0.81	1.4±0.29	1.2±0.04 *	1.4±0.18
TgMIC6	2.1±0.58	1.2±0.39	1.7±0.08	1.7±0.81
TgMIC1-4	6.3±1.2	2.2±0.9	1.0±0.1 *	2.0±0.15
TgMIC1-4-6	7.7±0.93 *	3.1±0.72 *	0.4±0.01 *	2.3±0.16*
Lac+	7.1±0.45 *	2.7±0.46 *	0.4±0.02 *	1.8±0.4

The results are expressed as the mean of five mice per group and are representative of three independent experiments. Statistical significance is denoted as * p < 0.05 compared to the PBS control group.

Serum concentrations of IL-12 and IFN-γwere significantly higher in animals from groups immunized with TgMIC1-4-6 and Lac+ (Table 1). IL-4 concentrations were significantly lower in the serum of all groups of immunized mice compared to that of the non-immunized mice, except in case of mice immunized with TgMIC6, which displayed IL-4 levels that were similar to the control group. Only mice immunized with TgMIC1-4-6 had IL-10 levels that were significantly higher than those in control mice.

### 3.5. Protection of immunized mice against *T*. *gondii* challenge

**Fig 5:**
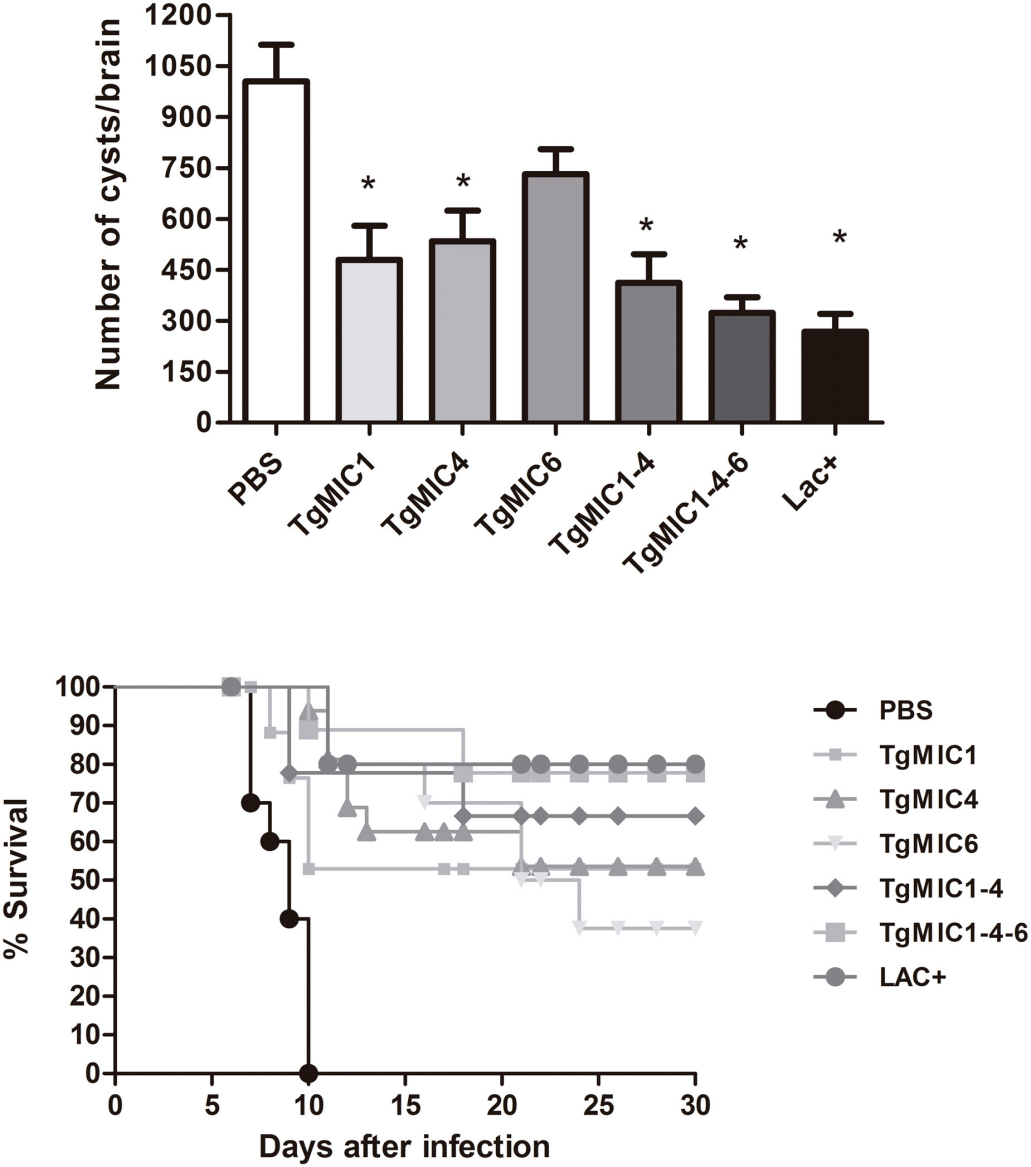
Number of brain cysts and survival of mice immunized with microneme proteins and infected with *T*. *gondii*. Mice immunized on days 0, 15, and 30 with the indicated preparations of TgMICs or with the vehicle (PBS) were challenged after one month (day 60) with *T*. *gondii* infection, provoked by gavage with cysts of the ME49 strain. (A) Cyst numbers were counted from whole brain homogenates of mice, harvested one month after challenge with 40 cysts. The results are expressed as means ± SD for each group. Significance is denoted as *p < 0.05, compared to the PBS group. (B) Survival curves of mice that were challenged with 80 cysts of the ME49 strain and observed daily for mortality. Data are representative of six experiments with similar results.

**Fig 6:**
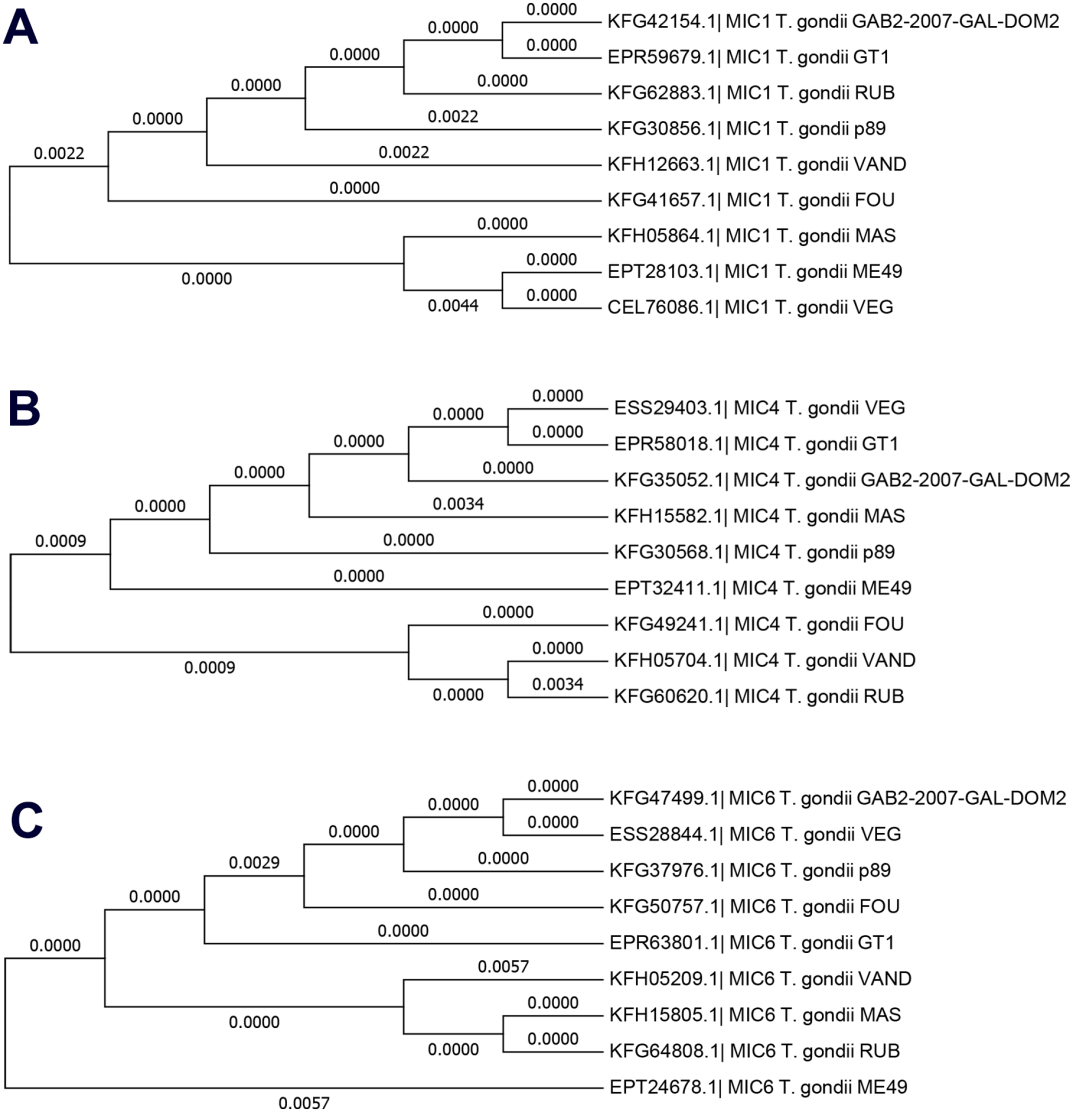
Cladogram analysis of TgMIC1, TgMIC4, and TgMIC6 of the *T*. *gondii* isolates. Dendrograms grouping microneme proteins of *T*. *gondii* isolates showing similarity of approximately 99% according to their sequences. (A) TgMIC1. (B) TgMIC4. (C) TgMIC6. Amino acids sequences were obtained from available sequence database at NCBI, and the alignment was performed using MEGA 6.06 software.

We evaluated, by enumeration of brain cysts, the effect of immunization in mice undergoing chronic toxoplasmosis. One month post the challenge, the mice immunized with TgMIC1, TgMIC4, TgMIC6, TgMIC1-4, TgMIC1-4-6, and Lac+, compared with non-immunized mice, showed reductions in brain cysts by 52%, 46.9%, 27.2%, 59%, 67.8%, and 73.4%, respectively (Fig 5A). The survival period (in days) of the mice challenged with *T*. *gondii* cysts is shown in Fig 5B. Non-immunized control mice started dying 6 days after *T*. *gondii* infection, and they were all dead by day 11 post infection. Effective and highly significant protection was demonstrated in mice immunized with TgMIC1-4-6 or TgMIC1-4, since 80% and 70% of the mice, respectively, survived to the acute phase of infection, which is compatible with the high protection reported to be conferred by immunization with the LAC+ fraction (21). Furthermore, immunization with individual components, namely, TgMIC1, TgMIC4, or TgMIC6, was associated with a survival rate of 40–50%, indicating that these vaccines confer partial protection against *T*. *gondii* infection. Since our results achieved significant protection in mice immunized with microneme proteins, as indicated above, we investigated whether this vaccine candidate could work also to different *T*. *gondii* strains. Alignment analysis demonstrated similarity approximately of 99% between TgMIC1 amino acid sequences from ME49 strain when compared to other strains (Fig 6A). Similar results were also verified to TgMIC4 (Fig 6B) and TgMIC6 (Fig 6C).

Together, our results show that the vaccine formulation constituted by the combination of three recombinant proteins (TgMIC1-4-6) induced the best immune response and subsequent protection against *T*. *gondii* infection.

## Discussion

In the current study, we investigated the protection conferred by immunization of C57BL/6 mice with several preparations of recombinant microneme proteins against *T*. *gondii* infection. The assayed preparations, with the exception of TgMIC6, conferred protection against infection. The most effective protection was conferred by the combination of TgMIC1, TgMIC4, and TgMIC6 (TgMIC1-4-6), as demonstrated by an increased survival rate and reduced tissue parasitism, which was associated with the occurrence of a Th1-specific immune response, the essential event in the control of toxoplasmosis.

The process of host cell invasion by *T*. *gondii* is complex and consists of several sequential steps initiated by the release of proteins from secretory organelles called micronemes (MICs) and rhoptries (ROPs) [25–27]. When released onto the parasite's surface, MICs can interact with host cell receptors, which lead to the activation of the gliding motility machinery, and finally culminate in host cell invasion [28–30]. Importantly, some MICs share adhesive domains, which are disposed in various combinations and numbers [16, 31–34]. Microneme protein 1 (TgMIC1), 4 (TgMIC4), and 6 (TgMIC6) form a complex on the surface of *T*. *gondii* and enable parasite binding to host cells [35]. TgMIC1 and TgMIC4 are both carbohydrate-binding proteins that interact with glycans containing terminal sialic acid and galactose residues, respectively, on host cells [18, 36–38]. Studies based on gene disruption have attributed a very important role for the TgMIC1-4-6 complex in host cell invasion in tissue culture and have established the contribution of the complex on parasite virulence *in vivo* [21, 39]. Although well known in terms of its structural features and adhesive properties, the role of TgMIC1-4-6 complex in host immunity is yet to studied in detail.

We have previously reported that mice immunized with the native subcomplex Lac+, comprising TgMIC1 and TgMIC4 proteins, elicit a strong specific immune response that confers protection against *T*. *gondii* infection [21]. In fact, due to the key biological role of micromeme proteins, some of them, including TgMIC11 [40], TgMIC13 [41], TgMIC8 [42], and TgMIC3 [43] have been proposed as vaccines for the prevention of toxoplasmosis. Thus, our goals in this work were to investigate the capacity of TgMIC1, TgMIC4, and TgMIC6 recombinant proteins of inducing protective immunity in mice against *T*. *gondii* infection.

To be efficient against toxoplasmosis, a vaccine should induce both cellular and humoral immune responses. Since a natural infection is often resolved by Th1-biased immunity [44, 45], a good vaccine must also direct T-helper cells toward the development of Th1 rather than Th2. Moreover, a humoral response is necessary because specific antibodies limit the multiplication of *T*. *gondii* by killing extracellular tachyzoites, either by activating the complement system or by opsonizing the parasites for phagocytosis and macrophage killing [46–51].

To evaluate whether prominent Th1 immunity was induced by the administration of microneme proteins, we first analyzed the isotype of serum-specific antibodies. All assayed preparations elicited both IgG1- and IgG2b-specific antibodies. The highest IgG2b/IgG1 ratio was detected in mice immunized with combinations of recombinant microneme proteins (TgMIC1-4, TgMIC1-4-6) or with the native complex Lac+ (isolated from STAg and comprising TgMIC1 and 4), suggesting that prominent Th1 immunity was elicited in these cases. This idea was strongly reinforced by the fact that the same groups of immunized mice had their spleen cells stimulated by STAg to proliferate and release Th1 cytokines, but not IL-4. Notably, high levels of IFN-γ were produced by mice immunized with TgMIC1-4-6. This is an important finding, considering that this cytokine plays an important role in host resistance to toxoplasmosis in its acute phase, by restricting growth of *T*. *gondii*, and later, by preventing the reactivation of parasites from dormant cysts [52–56]. The importance of IFN-γ in resistance to toxoplasmosis was unequivocally demonstrated by the extreme susceptibility of IFN-γ-deficient mice to *T*. *gondii* infection [55].

Pro-inflammatory responses, although essential for resistance to *T*. *gondii* infection, may lead to tissue injury if uncontrolled. The anti-inflammatory cytokine IL-10 acts by diminishing the detrimental effects of an excessive cellular immune response elicited during the acute phase of the infection [56–59]. Previous studies clearly demonstrated that IL-10-deficient mice rapidly succumb to infection by *T*. *gondii*, because of extensive necrosis of the liver, lungs, and intestines, which was attributed to an uncontrolled Th1 response [52, 60]. Therefore, we believe that the IL-10 production by spleen cells from immunized mice in our model was crucial for attenuating the Th1 immunity induced by *T*. *gondii* infection following vaccination.

We verified that vaccination with microneme proteins could positively influence the outcome of *T*. *gondii* infection, thus accomplishing the major purpose of the present work. The most successful vaccination procedure was the administration of the TgMIC1-4-6 preparation, since vaccinated mice had the highest survival rate (80%) and the lowest parasite burden in the brain. In addition, they displayed the most prominent Th1 immunity, which was equilibrated by IL-10 production. These results indicate that vaccination with the microneme complex TgMIC1-4-6 prevented host death in the acute phase of infection, conferring a protection that is reflected in the chronic phase of the disease. These results are particularly relevant if we consider that they were obtained in C57BL/6 mice, which are highly susceptible to *T*. *gondii* infection and a high mortality rate occurs during the acute phase of the infection even when animals are challenged with a low number of encysted bradyzoites [61].

In summary, the use of the multicomponent vaccine, comprising TgMIC1, TgMIC4, and TgMIC6, offers a promising strategy for conferring protection against toxoplasmosis. Evaluation in other animal host species, including those in which a vaccine may have veterinary utility, should aid in defining the applicability of this vaccine to prevent toxoplasmosis.
